# Studies in Clinical Medicine

**Published:** 1890-03

**Authors:** 


*Studies in Clinical Medicine.* By BYROM BRAMWELL, M.D., F.R.C.P. Ed., F.R.S.E. Nos. 1—18. Edinburgh: YOUNG J. PENTLAND. 1889–90.

This periodical, a record of some of the work done at Dr. Byrom Bramwell's Clinic in the Edinburgh Royal Infirmary, is issued fortnightly during the summer and winter sessions. It is a praiseworthy attempt by a physician eminently qualified for the task to preserve the best of the very useful work of an out-patient department. The cases are frequently well illustrated, and are recorded in Dr. Bramwell's well-known lucid style. The conversations in the Clinic are often given *verbatim*, and show the Scotch out-patient to be a far more pleasant person to deal with than his southern counterpart; for, without circumlocution, he gives just the answers his doctor requires, and in the fewest possible words.

Like Mr. Pentland's publications generally, this work is issued in an attractive form.

